# Prevalence of Helicobacter Pylori Infection in School and Pre-School Aged Children with C-14 Urea Breath Test and the Association with Familial and Environmental Factors

**DOI:** 10.4274/mirt.53215

**Published:** 2015-06-17

**Authors:** Alev Çınar, Murat Sadıç, Hasan İkbal Atılgan, Aylin Baskın, Gökhan Koca, Koray Demirel, Meliha Korkmaz

**Affiliations:** 1 Ankara Training and Research Hospital, Clinic of Nuclear Medicine, Ankara, Turkey; 2 Kahramanmaraş Necip Fazıl City Hospital, Clinic of Nuclear Medicine, Kahramanmaraş, Turkey

**Keywords:** Helicobacter pylori, C-14, urea breath test

## Abstract

**Objective:**

To investigate the prevalence of Helicobacter pylori (Hp) infection in pre-school and school age children with C-14 urea breath test, and to explore its association with age and socioeconomic factors in Turkey.

**Methods:**

Hp infection status was determined by using Urea Breath Test (UBT). Patients who had previous gastric surgery, Hp eradication treatment or equivocal UBT results were excluded. A questionnaire was administered to elicit information on gender, age, ABO/Rh blood group type, presence of gastric disease in the family, domestic animal in the household, and treatment for idiopathic Iron Deficiency Anemia (IDA).

**Results:**

This retrospective study included 500 pediatric patients (179 boys, 321 girls, mean age 10.7±4.3 years) of whom 62 (12.4%) were aged ≤6 years and 438 (87.6%) were aged 7 to 16 years. Helicobacter pylori (Hp) was positive in 245 (49%) cases. In the pre-school age group, 21/62 cases (34%) had positive UBT while in the school age group 224/438 children (51%) had positive UBT. A family history of dyspepsia and pet ownership were not associated with Hp positivity. Hp positive 76 (29.8%) children were on IDA treatment but this was not statistically significant.

**Conclusion:**

The Hp infection positivity rate was 49% in the pediatric age study group. The positivity rate was significantly lower at preschool age than school age, and it increased with age. There was no association with gender, ABO/Rh blood groups, presence of domestic pets, IDA, or history of gastric disease in the family.

## Abstract (tr)

**Amaç:**

Okul çağı ve okul öncesi yaş grubu çocuklarda C-14 Üre Nefes Testi (ÜNT) ile Helicobacter pylori (Hp) enfeksiyonunun prevalansını araştırmak ve Türkiye’de yaş ve sosyo-ekonomik faktörler ile ilişkisini ortaya çıkarmaktır.

**Yöntem:**

Hp enfeksiyonu varlığı ÜNT kullanılarak belirlendi. Daha öncesinde mide operasyon öyküsü olan, Hp eradikasyon tedavisi alan veya ÜNT değerleri şüpheli olan hastalar çalışmaya dahil edilmedi. Anamnez formu ile cinsiyet, yaş, boy, kilo, ABO/Rh kan grup tipi, ailede mide hastalığı öyküsü, evcil hayvan besleme ve demir eksiliği anemi tedavisi kullanımı öyküsü sorgulandı.

**Bulgular:**

Bu retrospektif çalışmaya 62’si (%12,4) ≤6 yaş, 438’si (%87,6) 7 ile 16 yaş aralığında olan 500 pediatrik hasta (179 erkek, 321 kız, ortalama yaş 10,7±4,3 yıl) dahil edildi. Hp 245 (%49) olguda pozitif bulundu. Okul öncesi yaş grubunda 21/62 olguda (%34) Hp ÜNT ile pozitif iken okul çağı grupta 224/438 (%51) çocukta Hp pozitif idi. Ailede dispepsi öyküsü ve evde evcil hayvan varlığı Hp pozitifliği ile ilişkili değildi. Hp pozitifliği demir eksikliği tedavisi alan 76 (%29,8) çocukta pozitif idi ancak bu istatistiksel olarak anlamlı değildi.

**Sonuç:**

Hp pozitiflik oranı pediatrik yaş grubunda %49 bulundu. Pozitiflik oranı okul öncesi çağda okul çağına göre anlamlı bir şekilde düşük iken bu oran yaş ile artmaktadır. Hp pozitifliği ile cinsiyet, ABO/Rh kan grubu, evcil hayvan besleme, demir eksikliği anemisi varlığı ve ailede mide hastalık öyküsü arasında ilişki bulunmamıştır.

## INTRODUCTION

Helicobacter pylori (Hp) is a well known cause of chronic active gastritis and plays an important role in the etiology of peptic ulcer in humans (1). It may occur heterogeneously at any age but once acquired, the infection may persist for years unless specific eradication therapy is provided. The transmission mechanism remains unclear. The prevalence of Hp infection is higher in developing countries than developed countries and in low socioeconomic groups (1,2).

It is currently estimated that approximately half the world’s human population is infected with this gastric bacterial pathogen, and its prevalence is thought to vary depending on patient’s chronological age, ethnic background, and socioeconomic conditions, as these are the most important factors influencing nutritional status and health during childhood (3,4).

Hp is associated with chronic gastritis and duodenal ulcers during childhood. As gastric ulcers are less common in children, eradication of Hp successfully reduces duodenal ulcer recurrence rate in infected children (4). Recent epidemiological studies have demonstrated the importance of intra-familial infection transmission, as well as transmission by contaminated water (4,5,6).

Several invasive and noninvasive methods are being used for the diagnosis of Hp infection in daily clinical practice. Invasive diagnostic methods requiring endoscopic biopsies with rapid urease testing, histological examination, and culture are still considered the gold standard when they are used in combination. Therefore, biopsy-based tests are recommended as the most reliable diagnostic method in children (7). As they are relatively invasive and costly, indirect tests can be preferred to detect Hp. Serum Hp-specific IgG antibodies can be tested with readily available commercial immunoassay kits, but these are not reliable for use in children, particularly in a setting of low prevalence. Hp specific antibody levels may be low especially in children younger than 10 years of age, which create false negative test results. In addition, both IgG and IgA subclasses continue to display false positive results for several months, even after Hp is eliminated from the host with specific eradication therapy (7,8).

Urea Breath Test (UBT) consistently produces better results when compared to other tests. UBT is performed by oral administration of C-13 or C-14 labeled urea to the patient. The urease enzyme that is produced by Hp metabolizes urea, if present in the stomach. The released radioactive 13CO2 or 14CO2 then diffuse in the blood, and is excreted into the expired air by the lungs. The radioactive carbon is then detected in breath (9).

Practice guidelines have been published for testing for Hp infection in children. All reports recommend Hp testing only when symptoms are sufficient to justify the potential risks of eradication therapy, and when a peptic ulcer is either proven or strongly suspected. Safety is the main issue regarding the application of UBT in children (10,11). The radiation equivalent of a 5 µCi (185 KBq) that is used in a standard UBT procedure is less than that of a chest X-ray. Attempts have been made to use lower doses of C-14 urea for UBT, especially in the younger population, and studies on the validation of microdose UBT for detection of Hp have revealed that this test provides high sensitivity and specificity (2,5,12,13,14,15,16,17,18,19,20). Microdose UBT with 1 µCi (37 KBq) is a simple, fast and inexpensive method with a negligible radiation burden for the diagnosis of Hp infection.

In this study, the aims were to investigate the prevalence of Hp infection in preschool and school age Turkish children by using the C-14 UBT, and explore its association with age, gender and environmental factors.

## MATERIALS AND METHODS

### Study Population

This retrospective study included 500 pediatric patients (179 male, 321 female, mean age 10.7±4.3 years, age range 3-16 years) who were referred to our nuclear medicine department for UBT between July 2007 and July 2013. Patients who had previous gastric surgery, Hp eradication treatment or those with equivocal UBT results (between 25 and 50 cpm) were excluded to prevent interference with the diagnostic accuracy of Hp infection. The Hp infection status was determined with UBT.

A standard questionnaire was prepared, and patients or their parents completed it. The assessments were based on these questionnaires. Information regarding gender, age, ABO/Rh blood group type, gastric disease in the family, domestic animal in the household, and treatment for IDA were extracted from the questionnaire.

Children were separated into two age groups as group 1 (preschool age-up to 6 years old), and group 2 (school age-7 to 14 years old) for statistical analysis. Patients who were receiving treatment for IDA were considered to have IDA.

### C-14 Urea Breath Test

After obtaining informed consent from all parents, a UBT was used to detect Hp infection of the gastric mucosa. None of the patients had undergone upper gastrointestinal operations or Hp eradication therapy. Antibiotics and bismuth compounds were discontinued for 4 weeks, H2 receptor antagonists and proton pump inhibitors were discontinued for 2 weeks, and anti-acids were stopped for at least 24 hours prior to the test. Patients were requested to fast overnight (at least 6 hours).

Capsules of urea (Helicap™, Isotop, Budapest, Hungary) labeled with 37 kBq (1 μCi) of C-14 were used for UBT. The patient swallowed the urea capsule with 50 ml of water. If Hp is positive in the stomach, urea is converted into C-14 labeled carbondioxide by urease enzyme, which is exhaled in the breath in 10 minutes. After 10 min, the patient was asked to inﬂate a balloon to obtain a breath sample (Heliprobe BreathCard™, Kibion, Stockhom, Sweden) until the indicator membrane changed color from orange to yellow. The activity was counted for 250 seconds and the results were given in counts per minute (cpm) by a Heliprobe analyzer (Heliprobe™-analyzer, Kibion, Stockhom, Sweden), a special Geiger-Müller counter. Samples with values ≤25 cpm were considered negative for Hp, samples with values between 26 and 49 cpm were considered suspicious, and those with values ≥50 cpm were considered positive for Hp infection.

### Statistical Analysis

Statistical analyses were performed using SPSS software version 15. The proportions of patients with positive test results were presented with gender, blood type, gastric disease in the family, pet in the household, IDA presence using cross-tabulations. The Chi-square test or Fischer’s exact test, where appropriate, was used to compare these proportions in different groups. A p-value of <0.05 was considered statistically significant.

## RESULTS

The study included 500 children; 321 (64.2%) were girls and 179 (35.8%) were boys. Out of the total 500, 62 (12.4%) were ≤6 years of age and 438 (87.6%) were between 7 and 16 years of age. Hp was positive in 245/500 (49%) cases. In the pre-school age group, 21/62 cases (33.9%) were positive for Hp, and in the school-age group 224/438 children (51.1%) had a positive UBT. In 84/179 (46.9%) boys the test was positive, while 161/321 (50.2%) girls were positive for Hp. In this study, there was no statistically significant difference between genders for UBT positivity (p=0.400), but a statistically significant association was found with age (Table 1). UBT positivity was seen significantly more in school-age children (p=0.011) (Table 1).

A family history of dyspepsia was present in only 12.9% of cases, but there was no statistically significant association with Hp prevalence (p=0.77). The number of children with pets at home was 11 (4.3%) and this was not associated with Hp positivity (p=0.39). Seventy-six (29.8%) of Hp positive children were receiving iron deficiency anemia treatment, however, this was not statistically significant (p=1.00).

Table 2 shows the proportions of patients with Hp positive test results presented by blood type, presence of gastric disease in the family, pet in the household and IDA.

## DISCUSSION

Within the array of non-invasive tests that are used to diagnose active Hp infection in children, UBT detects urease activity with both high sensitivity and high specificity. However, oral urease-producing bacteria may cause false positive results. The advantage of this test is that it can be performed easily and the result is known within 10 minutes (21,22,23). C-14 is a low energy beta-emitter (49 keVmean, 156 keVmax) with maximal range in water of 0.3 mm, and a physical half-life of 5730 years that reaches maximum activity in the exhaled air after 10-15 min, decreasing over time with a biological half-life of 15 min. C-14 urea, which is not broken down, is excreted in the urine. With t½=12 h, while 10% of C-14 remains in the body after 72h, C-14 urea that is not metabolized by bacteria is eliminated in the urine. The labeled CO2 is absorbed into the blood and exhaled in the breath. Samples of the excreted CO2 are collected, and then analyzed for the presence of the isotope.

A high prevalence of Hp infection in children has been reported several times in developing countries in contrast to developed ones. In the developing world, its prevalence among patients with dyspepsia is 80% to 90%, and the annual incidence of new infections is 3 or more per 100 susceptible persons (13). A retrospective multicenter study in Japan demonstrated an association between Hp infection and the development of gastric ulcers in children, and reported that most children with Hp infection are asymptomatic without any disease sequel (14). In the current study group, the main symptom in children who were found to be Hp positive was poor appetite.

Hp infection is associated with iron deficiency and growth disturbances in children and many studies have supported the view that Hp has a role in the development of refractory IDA (14,15). A prospective open study demonstrated that 92% of adults recover from anemia, showing increased serum ferritin levels, following eradication of Hp infection. Hp has an impact on hematological outcomes, and the prevalence of iron deficiency and IDA is reported to be lower in Hp negative children as compared to Hp positive children (24,25). On the other hand, three studies found no association between Hp infection and IDA (16). In the current study, no statistically significant association was determined between IDA and Hp positivity.

The children of Hp infected mothers have been reported to have a higher risk of acquiring Hp infection (26). Such a risk is further increased if the siblings are infected, and the risk of acquiring infection before 6 years of age is significantly increased when the child’s mother tests positive for infection. The close contact with the mother could explain the increased Hp prevalence in children below 6 years of age, especially when the mother has the infection. In the current study group, no association was found between presence of dyspeptic family members and Hp positivity in children.

Parallel to results of a EUROGAST study on the risk factors for Hp, Milroad et al. evaluated association of Hp with blood type, Rh factor and gender on 227 patients with gastric biopsy and did not find a statistically significant difference (27,28). Similar to the results of that study, a statistically significant association was not detected between Hp prevalence and gender or blood type in the current study.

## CONCLUSION

The present study provided useful data on Hp status with C-14 urea breath test, in school and preschool aged Turkish children, and its association with familial and environmental factors. As a result, we think that while investigating Hp on the patients with childhood age, further studies to investigate this relationship are warranted.

## Figures and Tables

**Table 1:**
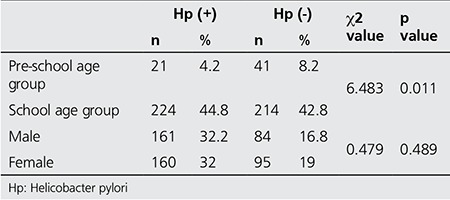
The association of Helicobacter pylori (Hp) positivity with age groups and gender

**Table 2:**
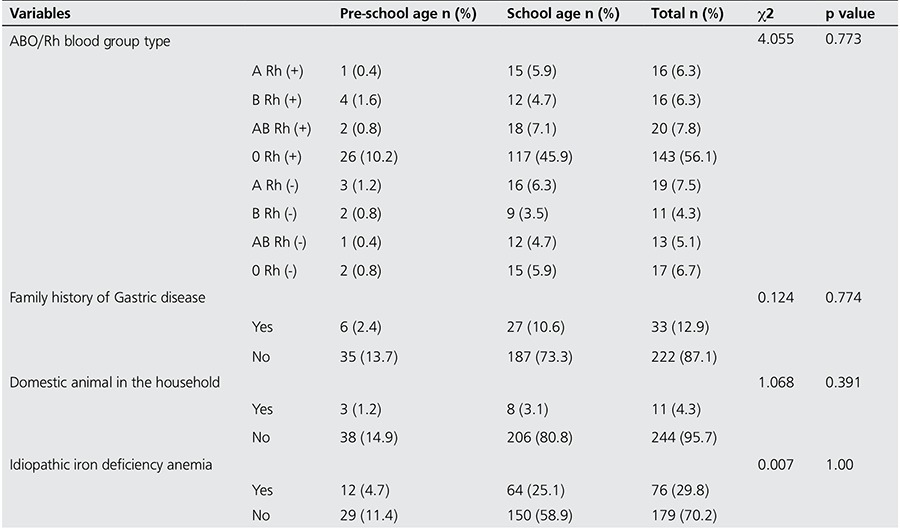
Patients with positive test results presented by blood type, presence of gastric disease in the family, domestic animal in the household and idiopathic iron deficiency anemia
